# Flux 2 Editorial

**DOI:** 10.1016/j.dcn.2016.03.003

**Published:** 2016-03-04

**Authors:** Dima Amso, Bradley Schlaggar

It has been a pleasure to edit this special issue for *Developmental Cognitive Neuroscience*. The issue is a showpiece of the scientific contributions made at the second Flux International Society for Integrative Developmental Cognitive Neuroscience meeting held in 2014 in Hollywood, CA. Flux is quickly becoming the favorite conference of developmental cognitive neuroscientists, where we can engage and learn from each other in an encouraging and exciting scientific atmosphere.

One of the stated purposes of the Flux Society and its annual conference is to *promote public information by discussing implications on the fields of education, health, juvenile law, parenting, and mental health, and to encourage further progress in the field of developmental cognitive neuroscience*. The papers in this special issue offer us the opportunity to commend this *avante-gard* aspect of the field of developmental cognitive neuroscience. Ours is a unique scientific endeavor with a social conscience and an eye toward policy. Papers in this special issue are rigorous, detailed, and interdisciplinary in that they take seriously the responsibility to affect change through science. Topics covered include how best to train executive functions, the impact of early maternal separation on subsequent cognitive flexibility, the role of socioeconomic status in attention and memory development in infancy, and the circuitry underlying major depressive disorder in children.

Indeed, the papers in this special issue represent the best of empirical studies, theoretical contributions, and methodological advances. Collectively, the contributions cover the developing child in infancy, in childhood, and through to adolescence. We extend a special thank you to our contributors for their excellent pieces and to Elsevier and *Developmental Cognitive Neuroscience*, the official journal of Flux.

